# Clinical application of CMR in cardiomyopathies: evolving concepts and techniques: A position paper of myocardial and pericardial diseases and cardiac magnetic resonance working groups of Italian society of cardiology

**DOI:** 10.1007/s10741-022-10235-9

**Published:** 2022-05-10

**Authors:** Marco Merlo, Giulia Gagno, Anna Baritussio, Barbara Bauce, Elena Biagini, Marco Canepa, Alberto Cipriani, Silvia Castelletti, Santo Dellegrottaglie, Andrea Igoren Guaricci, Massimo Imazio, Giuseppe Limongelli, Maria Beatrice Musumeci, Vanda Parisi, Silvia Pica, Gianluca Pontone, Giancarlo Todiere, Camilla Torlasco, Cristina Basso, Gianfranco Sinagra, Pasquale Perrone Filardi, Ciro Indolfi, Camillo Autore, Andrea Barison

**Affiliations:** 1 Cardiothoracovascular Department, Azienda Sanitaria Universitaria Giuliano Isontina (ASUGI), University of Trieste, Trieste, Italy; 2 Cardiology, Department of Cardiac Thoracic Vascular Sciences and Public Health, University of Padova, Padova, Italy; 3 Cardiology Unit, St. Orsola Hospital, IRCCS Azienda Ospedaliero-Universitaria Di Bologna, 40138 Bologna, Italy; 4 Cardiologia, IRCCS Ospedale Policlinico San Martino, Genova, Italy; 5 Dipartimento di Medicina Interna e Specialità Mediche, Università degli Studi di Genova, Genova, Italy; 6 Department of Cardiology, IRCCS Istituto Auxologico Italiano, Milan, Italy; 7 Division of Cardiology, Ospedale Accreditato Villa dei Fiori, 80011 Acerra, Naples, Italy; 8 University Cardiology Unit, Department of Emergency and Organ Transplantation, University of Bari, 70124 Bari, Italy; 9 Cardiothoracic Department, University Hospital “Santa Maria Della Misericordia”, Udine, Italy; 10 Inherited and Rare Cardiovascular Disease Unit, Department of Translational Medical Sciences, University of Campania “Luigi Vanvitelli”, AORN Dei Colli, Monaldi Hospital, Naples, Italy; 11 Cardiology, Clinical and Molecular Medicine Department, Faculty of Medicine and Psychology, Sapienza University of Rome, 00189 Rome, Italy; 12 Multimodality Cardiac Imaging Section, IRCCS Policlinico San Donato, Milan, Italy; 13 Dipartimento di Cardiologia Perioperatoria e Imaging Cardiovascolare, Centro Cardiologico Monzino IRCCS, Milan, Italy; 14 Fondazione Toscana Gabriele Monasterio, Pisa, Italy; 15 Dipartimento Scienze Biomediche Avanzate, Università degli Studi Federico II, Mediterranea CardioCentro, Naples, Italy; 16 Dipartimento di Scienze Mediche e Chirurgiche, Cattedra di Cardiologia, Università Magna Graecia, Catanzaro, Mediterranea Cardiocentro, Napoli, Italy

**Keywords:** Cardiac magnetic resonance, Cardiomyopathies, Diagnosis, Prognosis

## Abstract

Cardiac magnetic resonance (CMR) has become an essential tool for the evaluation of patients affected or at risk of developing cardiomyopathies (CMPs). In fact, CMR not only provides precise data on cardiac volumes, wall thickness, mass and systolic function but it also a non-invasive characterization of myocardial tissue, thus helping the early diagnosis and the precise phenotyping of the different CMPs, which is essential for early and individualized treatment of patients. Furthermore, several CMR characteristics, such as the presence of extensive LGE or abnormal mapping values, are emerging as prognostic markers, therefore helping to define patients’ risk. Lastly new experimental CMR techniques are under investigation and might contribute to widen our knowledge in the field of CMPs. In this perspective, CMR appears an essential tool to be systematically applied in the diagnostic and prognostic work-up of CMPs in clinical practice. This review provides a deep overview of clinical applicability of standard and emerging CMR techniques in the management of CMPs.

**Supplementary information:**

The online version contains supplementary material available at 10.1007/s10741-022-10235-9.

## Introduction

Cardiomyopathies (CMPs) are a group of myocardial disorders, often affecting young individuals, characterized by the presence of structural and functional abnormalities of the heart muscle, not explained by coronary artery disease, hypertension, valvular disease or congenital heart disease [1]. Advancements in medical treatments and the availability of implantable cardioverter defibrillator to prevent sudden cardiac death (SCD) have allowed a substantial increase in the survival of affected individuals, thus making early diagnosis and prompt treatment mandatory [2].

The non-invasive characterization of cardiomyopathies has received a great boost from the recent advances in cardiovascular magnetic resonance imaging (CMR), which to date represents the gold standard for non-invasive assessment of cardiac morphology, function and myocardial tissue changes. In fact, CMR allows not only the quantification of biventricular volumes, mass, wall thickness, systolic- and diastolic function, intra- and extracardiac flows, but also the detection of myocardial oedema, fibrosis, and the accumulation of other intra/extracellular substances (such as fat, iron, amyloid), providing unique information for the etiological, diagnostic and prognostic definition of the disease. In addition to the conventional sequences, new quantitative techniques are now available and further experimental CMR techniques are under investigation and might contribute to widen our knowledge in the field of CMP. The purpose of this joined document of Working Groups on Myocardial and Pericardial Diseases and on CMR of Italian Society of Cardiology is to provide practical information for the application of both standard and emerging CMR techniques in the clinical management of CMPs, bringing the most recent scientific evidence to daily clinical practice.

## Overview of CMR sequences in cardiomyopathies (Table 1 and Figure 1)

**Table 1:** Overview of most common CMR sequences

	Sequence characteristics	Applications	Limitations
***Native imaging (without contrast injection)***			
Cine imaging	Balanced-SSFP (segmented, ECG-gated, multiple cardiac phases) *Possible 3D acquisition (but lower spatial/temporal resolution, longer acquisition times)*	LV/RV volumes, systo/diastolic function, wall thickness, LV/RV mass Banding (“India Ink”) artifacts highlight fat/water boundaries (e.g. fat infiltration)	Susceptibility to magnetic field inhomogeneities (e.g. metal implants, poor shimming) Acquired over multiple heartbeats (limited by irregular RR-intervals or breathing movements) Lower temporal resolution than echocardiography
	Spoiled-GRE (segmented, ECG-gated, multiple cardiac phases)	Used in case of metal implants (lower susceptibility to metal artifacts)	Lower contrast (blood-to-myocardium) resolution Limited by arrhythmias/ breathing movements (similarly to SSFP cine)
	Real-time GRE or SSFP (single-shot, ungated, multiple cardiac phases)	Used to track beat-to-beat cardiac motion (e.g. septal movements in suspected tamponade/ constrictive physiology; diaphragmatic movements in suspected paralysis)	Low spatial and temporal resolution
Black-blood imaging	T1- or PD- or T2-weighted double-IR FSE (segmented or single-shot, ECG-gated, triggered to a single diastolic cardiac phase)	LV/RV morphology and tissue-characterization (e.g. fatty infiltration) T2-weighted fat-saturated IR-FSE used as an alternative to T2-weighted STIR sequences for oedema detection	Still fluids (subendocardial bloodpool, effusions…) appear hyperintense
STIR	T2-weighted triple-IR FSE (segmented or single-shot, ECG-gated, triggered to a single diastolic cardiac phase)	Intra/extracellular oedema, such as in inflammation and acute necrosis (qualitative/ semiquantitative detection of hyperintense areas) Markedly hypointense areas correspond to myocardial haemorrage or calcifications	Quantification of oedema is time consuming Cardiac segments close to the surface coil may appear hyperintense Still fluids (subendocardial bloodpool, effusions…) appear hyperintense
T1-mapping	MOLLI (8–11 single-shot, ECG-gated IR-SSFP images, all acquired at the same systolic or diastolic cardiac phase with different TIs) Other IR- or SR- sequences are possible alternatives	Native T1 (quantitative): increased by inflammation, oedema, vasodilation, fibrosis, amyloid; decreased by fat, iron	Limited spatial resolution Needs motion correction algorithms (image misalignment may cause incorrect T1 calculation)
T2-mapping	MESE (Multi echo spin echo), GraSE (Gradient echo spin echo) or T2-prepared bSSFP: 3–4 images, all acquired at the same systolic or diastolic cardiac phase with different T2-weighing	Native T2 (quantitative); increased by inflammation, oedema; decreased by iron	Limited spatial resolution Needs motion correction algorithms (image misalignment may cause incorrect T1 calculation)
T2*-mapping	GRE multiecho: 6–8 segmented, ECG-gated images, all acquired at the same systolic or diastolic cardiac phase with different T2*-weighing	Native T2* (quantitative); decreased by iron deposition (haemochromatosis, haemorrage)	Limited spatial resolution Susceptibility to magnetic field inhomogeneities (e.g. metal implants, poor shimming)
Phase contrast	Spoiled-GRE (segmented, ECG-gated, multiple cardiac phases) *Possible 3D/4D acquisition (but longer acquisition times and motion artifacts)*	Flow quantification (quantitative), across cardiac valves, aortic or pulmonary vessels	Limited spatial and temporal resolution compared to Doppler-echocardiography Unsuitable for vessels as small as the coronary arteries Inaccurate in case of magnetic field inhomogeneities
***Post-contrast imaging (after Gd-based contrast injection)***			
Perfusion	IR- or SR-, GRE or SSFP during Gd-based contrast injection	Myocardial perfusion (qualitative/semiquantitative) Quantitative myocardial perfusion with specific dual-bolus or dual-sequence techniques	Limited spatial resolution Possible dark rim artifact in the subendocardial blood-to-myocardium interface
Early enhancement (EGE)	IR GRE or IR-SSFP (segmented or single-shot, ECG-gated, triggered to a single systolic/diastolic cardiac phase), with a TI set to null the thrombus	Intracardiac thrombus detection	Selection of a wrong nulling time makes EGE image inaccurate
Late enhancement (LGE)	IR GRE or IR-SSFP (segmented or single-shot, ECG-gated, triggered to a single systolic/diastolic cardiac phase), with a TI set to null the normal myocardium *Possible 3D acquisition (but longer acquisition times and more motion artifacts)*	Extracellular Gd deposition (qualitative/semiquantitative, increased by necrosis, fibrosis, amyloid deposition but also intense extracellular oedema) with excellent contrast-to-noise ratio Markedly hypointense areas within LGE correspond to no-reflow areas	Quantification of fibrosis is time consuming Detection of diffuse fibrosis remains challenging Selection of a wrong nulling time makes LGE image inaccurate; PSIR (phase sensitive inversion recovery) LGE less dependent on TI
ECV-mapping	Same as T1 mapping (MOLLI or other sequences)	Extracellular Gd deposition (qualitative): increased by necrosis, amyloidosis, amyloid, but also extracellular oedema	Limited spatial resolution Needs a pre- and post-contrast acquisition, with perfect image fusion Needs blood haematocrit for ECV calculation
**Vascular imaging**			
CEMRA	3D GRE during Gd-based contrast injection *Possible time-resolved CEMRA acquisition (but lower spatial resolution)*	Aorta and its branches, pulmonary arteries and its branches,	Needs contrast injection ECG-ungated (unsuitable for coronary arteries) Lower spatial resolution than CT
3D-whole heart	3D balanced-SSFP (segmented, ECG-gated, respiratory navigator- gated, triggered to a single cardiac phase)	Coronary artery anatomy Cardiac arterial and venous connection anatomy	Long acquisition time Limited by arrhythmias/ breathing movements

Common CMR sequences are based on an FSE, spoiled-GRE or a SSFP structure (readout), with variable T1/PD/T2 weighing depending on the chosen parameters (flip angle, repetition time, echo time), sometimes preceded by an IR- or SR- prepulse (to selectively invert or saturate specific tissues)

*CEMRA*, contrast-enhanced magnetic resonance angiography; *CT*, computed tomography; *ECV*, extracellular volume; *FSE*, fast spin-echo; *GRE*, gradient echo; *IR*, inversion recovery; *LV*, left ventricle; *MOLLI*, modified Look-Locker inversion recovery; *PD*, proton density; *RV*, right ventricle; *SR*, saturation recovery; (b) *SSFP*, (balanced) steady-state free-precession; *STIR*, short-tau inversion recovery; *TI*, inversion time

**Fig. 1:**
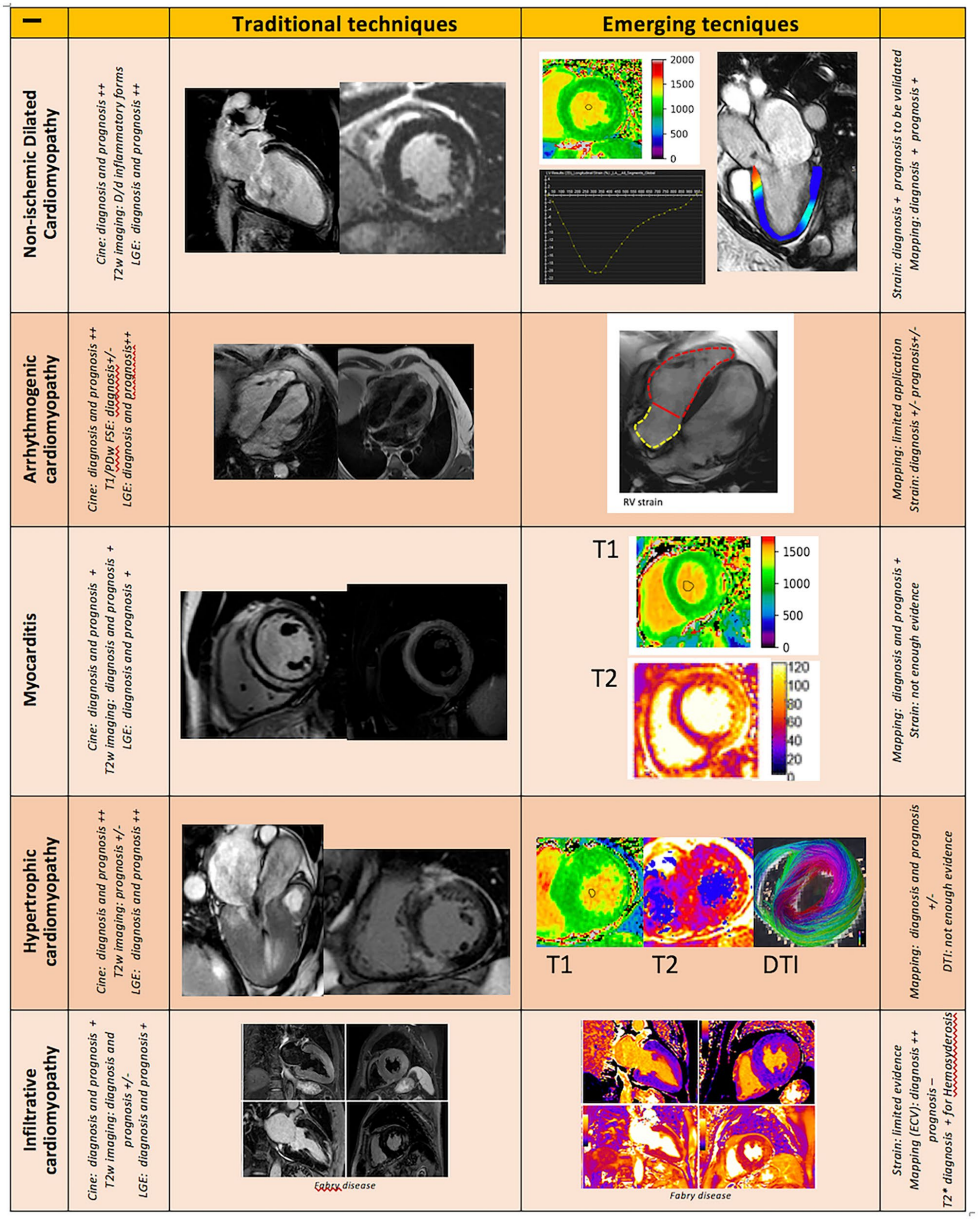
Main standard and emerging CMR techniques and their main application in the diagnostic and prognostic work up of several cardiomyopathies. Legend: “ + + ” very useful, “ + ” useful, ± “not so useful”, “- “ not useful

CMR is a multiparametric, highly reproducible, non-invasive imaging technique, with a relatively high spatial, temporal and contrast resolution [3–5]. This is made possible thanks to a great number of different sequences, each obtained combining specific magnetic gradients and radiofrequency pulses, whose detailed explanation goes beyond the scope of this review (for detailed description see Table 1 and Fig. 1).

The most common conventional sequences in CMR are cine steady state free-precession (SSFP) images for the assessment of cardiac volumes, wall thickness, mass and systolic function [6] and several different static sequences for myocardial tissue characterization. For instance, fatty infiltration can be seen as a dark “India Ink” sign in SSFP images or as a hyperintense area in T1 or PD-weighted fast spin echo (FSE) sequences [7] while myocardial edema appears hyperintense in T2-STIR (short-tau inversion-recovery) sequences. Fibrosis can be seen as a hyperintense area on late gadolinium enhancement (LGE) sequences, which are acquired 10–15 min after gadolinium-based contrast agent administration. The various pattern of LGE have been used to distinguish ischemic cardiomyopathy (characterized by subendocardial or transmural LGE, corresponding to a coronary territory) from primary nonischemic cardiomyopathies (characterized by patchy or mid-wall LGE), myocarditis (sub-epicardial LGE) and cardiac amyloidosis (diffuse subendocardial-to-transmural LGE).

As compared to the wide range of information derived from CMR, there are only few contraindications, mostly related to MR-unsafe metal implants, severe renal failure (which limits the use of several gadolinium-based contrast agents), patient discomfort (claustrophobia) and tachyarrhythmias or poor breath-holding (with consequent impairment of image quality) [8, 9].

Compared to conventional imaging, the novel mapping sequences allow the absolute quantification of T1, T2, and T2* relaxation times (ms) for each tissue generating pixel-wise quantitative myocardial maps [10, 11], reflecting changes due to several myocardial diseases [12].

Native (pre-contrast) T1 mapping encompasses both intracellular and extracellular changes: myocardial infarction, inflammation, edema, fibrosis or amyloid all demonstrate prolonged native T1 values compared with normal myocardium, while iron (in cardiac hemochromatosis) or lipids (as in Fabry disease) shorten pre-contrast T1 [12, 13].

- The myocardial extracellular volume (ECV) is calculated from pre- and post-contrast T1 mapping and hematocrit and correlates with the extent of interstitial space (where gadolinium-based contrast agents accumulate). Myocardial necrosis, interstitial oedema, fibrosis and amyloidosis are the most common causes of an increased ECV [14, 15]. Differently from LGE, ECV mapping does not require the presence of local differences in the myocardium, thus allowing the detection of diffuse myocardial changes (i.e. diffuse interstitial fibrosis), which can hardly be detected with the sole LGE technique.
- T2 mapping detects myocardial oedema, with a higher sensitivity and reproducibility than T2-STIR sequences [16], in both ischemic and non-ischemic cardiac diseases.
- T2* differs from T2 mapping because it accounts for magnetic field inhomogeneities, and it has emerged as a valuable tool in the detection and quantification of myocardial iron deposits, such as in myocardial hemorrhage and hemochromatosis [17, 18].

Further experimental CMR techniques (resumed in Supplemental Table 1) are under investigation and may become available for clinical practice in the near future.

## Non-ischemic dilated cardiomyopathies

Non-ischemic dilated cardiomyopathy (DCM) is characterized by the presence of a poorly contractile and frequently dilated left and/or right ventricle, resulting from a complex interplay between individual genetic background and environmental factor [19].

In this context, CMR is now acknowledged as the gold standard technique for the quantification of chamber volumes, mass, and ejection fraction (EF) [20, 21]. Furthermore, CMR has the ability to characterize myocardial tissue and to detect myocardial fibrosis, which has been recognized to have a prognostic relevance in patients with DCM, thus improving risk stratification and patients’ outcome. Therefore, it is widely accepted that all DCMs should undergo an early CMR as a part of the diagnostic and prognostic workup.

Histological studies have pointed out that in DCM fibrosis can occur in two forms [22]. One is irreversible replacement fibrosis, corresponding to the presence of LGE, which depicts areas of myocardial scarring developed as a consequence of cell death [22, 23]. LGE can be found in about 30–40% of DCM patients, the most typical pattern being in the midwall of the interventricular septum, even if also a subepicardial pattern can be found, especially in post inflammatory DCM [24]. Since the first prospective longitudinal study conducted in 2006 by Assomull et al. [25], midwall fibrosis detected by LGE has emerged as a predictor of adverse prognosis in patients with DCM, including all-cause mortality, hospitalization and SCD/VT. Subsequent studies have confirmed these data, pointing out that the presence of myocardial scar allows to identify a subgroup of patients at a higher risk of adverse outcome independently from LVEF [24, 26]. A recent meta-analysis [24], confirmed that the presence of LGE is significantly associated with arrhythmic endpoint, such as SCD, sustained VT and appropriate ICD therapy (pooled OR 4.3, 95% CI 3.3 to 5.8, *p* = 0.001). Moreover, in this meta-analysis LVEF was not able to predict arrhythmic events in DCM, while a significant association between LGE and VA or SCD was observed also in patients with LVEF above 35%. On these bases, the recently published ESC guidelines [20], which have reduced ICD recommendation class for patients with non-ischemic CMP and severely reduced EF (i.e. class IIA, level of evidence A), encompass the use of LGE as a tool with additional value to LVEF for the identification of the best candidates to ICD implantation in primary prevention [26, 27]. However, no specific cut off have been validated and patients should be counseled on individual basis. Furthermore, whether LGE localization, pattern of distribution or LGE extension could have a prognostic impact is still not clear and further investigations are needed. CMR could also be useful in patients receiving cardiac resynchronization therapy (CRT) thanks to its capability to guide LV lead placement away from scarred tissue [28, 29].

The second form of fibrosis is interstitial and it is due to the accumulation of collagen even in the absence of cell death [30]. This form of fibrosis may be detected and quantified by native myocardial T1 relaxation times and ECV, and it has recently emerged as an independent marker of poor outcome [31–33].

CMR can be a valuable tool also in the analysis of right ventricle, often poorly visualized by echocardiography, which has emerged as an important tool in DCM risk stratification [34].

Finally, another promising CMR derived parameter is represented by global longitudinal strain (GLS) measured by feature-tracking analysis which was found to correlate better than LVEF and BNP with the composite of cardiac death, heart transplantation and appropriate ICD shock due to VT or VF, in a DCM population [35, 36].

## Arrhythmogenic cardiomyopathy

Arrhythmogenic cardiomyopathy (ACM) is a genetically-determined heart muscle disease characterized by fibro-fatty myocardial replacement, clinically associated with malignant ventricular arrhythmias and SCD [37]. Although originally described as a disease with predominant right ventricular (RV) involvement, subsequent increasing recognition of biventricular and left dominant phenotypic variants has led to broad the concept of arrhythmogenic cardiomyopathy as a disease potentially involving both right and left ventricles [38].

CMR has always been considered as a non-invasive tool for the demonstration of morpho-functional abnormalities. In the recently published “Padua Criteria” [39] CMR has gained further importance. In fact, while according to the previous diagnostic criteria the presence of structural myocardial abnormalities could only be detected by endomyocardial biopsy, it is now contemplated to detect these abnormalities also with CMR (LGE). Accordingly, it is now mandatory to perform CMR in patients with known or suspected ACM.

The T1 weighted images, once considered useful to identify fatty infiltration, have limited sensitivity and specificity because of poor resolution and partial volume artifacts [40–42] and might be replaced by the detection of “India Ink” artifacts in conventional cine-SSFP images [7]. The routine use of T2-weighted images for the depiction of myocardial edema is also not recommended, unless in case of “hot-phase” presentation (chest pain and troponin release), which are common for instance in pediatric patients and carriers of desmoplakin gene mutations [43]. It is instead mandatory to acquire LGE images which allows the detection of areas of fibro-fatty myocardial replacement, that are the hallmark lesions of ACM and which adds valuable information for arrhythmic risk stratification, particularly in left-dominant forms [44]. In RV diseases, LGE assessment can be challenging and limited by a high intra-interobserver variability; however, when considered together with wall motion abnormalities, it increases CMR accuracy for the diagnosis of ACM [45]. In LV arrhythmogenic diseases, LGE is commonly found in the subepicardial layers of the LV free wall, especially in the inferolateral region, with or without septal involvement [42]. The presence of circumferential LV subepicardial LGE in short axis view (“ring pattern”) has been consistently reported in left-dominant variants with specific genotype [46, 47]. As in DCM, it is clearly the emerging impact of CMR (and specifically LGE) on top of standard risk scores to identify high arrhythmic risk patients, candidates to primary prevention ICD implantation when a LV dominant form is present, regardless the amount of systolic dysfunction [44].

The new CMR techniques, such as T1 and T2 mapping, still have limited applications in patients with ACM. Conversely, feature-tracking CMR has recently raised interest given its potential capability to detect subtle segmental impairment of wall contraction, useful to early identify ACM patients in concealed phases of disease, as well as family members and asymptomatic gene carriers [48]. Supplementary materials, case 1.

## Acute myocarditis

Acute myocarditis (AM) is an inflammatory disease of the myocardium with different aetiology and with a heterogeneous presentation and clinical course that make patients’ management and risk stratification challenging. [49]. The diagnosis of AM can be confirmed only when histological Dallas Criteria are met, being therefore endomyocardial biopsy (EMB) necessary. Despite being an invasive examination with potentially life-threatening complications, EMB is indicated in selected myocarditis patients with hemodynamic instability not responsive to conventional medical treatment as well as when specific myocarditis aetiologies are suspected, also in hemodynamically stable patients [20, 50]. The limited availability of EMB has been compensated for by the increased use of CMR, which is able to characterize myocardial tissue and to identify areas of myocardial oedema and fibrosis/necrosis, thus allowing a non-invasive diagnosis of AM.

According to the original Lake Louise Criteria (LLC) the diagnosis of myocarditis could be made in the presence of “any 2 out of 3” CMR markers, consisting of T2-weighted, Early Gadolinium Enhancement and Late Gadolinium Enhancement (LGE) sequences, assessing myocardial edema, hyperemia and fibrosis/necrosis, respectively [51]. LLC have been shown to be very sensitive in the diagnosis of AM in patients presenting with chest pain, while sensitivity was reduced in those presenting with arrhythmias or heart failure [52].

The advent of parametric mapping has allowed overcoming some of the limitations of standard T2-weighted and T1-weighted sequences. In fact, each tissue has a characteristic range of T1 and T2 values which are altered in case of increase in the free water content (such as in myocardial inflammation) [10, 53].

Consequently, the LLC criteria have been recently updated so that, in order to achieve the diagnosis of AM, it is now necessary the presence of both a “T1 criterion” (presence of LGE, increased native T1-mapping or extracellular volume values) and a “T2 criterion” (hyperintensity on T2 weighted sequences or increased T2 mapping values) [54].

While T1 mapping and ECV seem to be altered both in acute as well as chronic myocarditis, T2 mapping has proved to be better correlated with the disease activity (inflammation), thus allowing the detection of AM and its differentiation from chronic inflammation with better accuracy [55].

Although limitations for the applicability of parametric mapping still exist (i.e. the lack of universal reference values), the evaluation of native T1 and T2 mapping, has been shown to led to an increase in CMR diagnostic accuracy, therefore advanced tissue characterization comprehensive of T1 and T2 mapping is now highly recommended by international consensus in all patients with suspected myocarditis, whenever feasible. [3, 56]

**Fig. 2:**
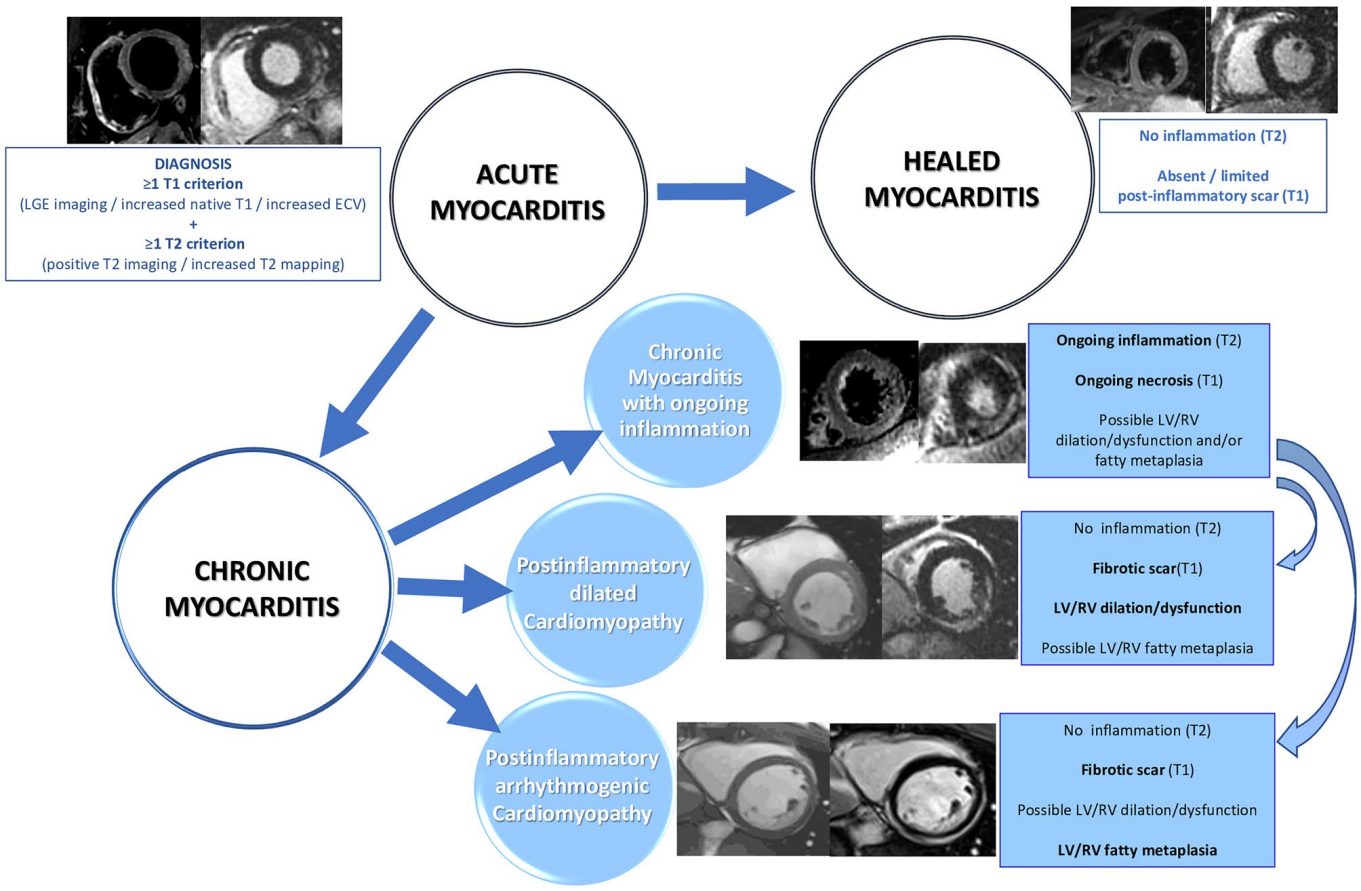
Useful of standard and emerging CMR techniques in the natural history of inflammatory cardiomyopathy. Legend: “RV” right ventricle, “LV” left ventricle

Apart from the role of CMR in the diagnosis of AM, several studies have investigated the potential contribution of tissue characterization by CMR in patients’ risk stratification. While a normal CMR correlates with a favorable outcome, several studies have confirmed the negative prognostic value of LGE as well as the correlation between abnormal T2-weighted imaging and worse outcome [57, 58]. Feature tracking analysis, thanks to a better assessment of systolic function and LV kinetic, has already demonstrated both to be helpful in detecting AM with preserved ejection fraction, and to be promising tool in patients’ risk stratification, even if more studies are needed to confirm these preliminary data [59–61] Fig. 2.

## Hypertrophic cardiomyopathy

Hypertrophic cardiomyopathy (HCM) is a genetic disease characterized by inappropriate hypertrophy, myocardial fibrosis and diffuse disarray with diverse phenotypic expressions, clinical course and prognosis [62].

Cardiovascular magnetic resonance (CMR) is capable to provide assessment of ventricular mass, chamber volume, cardiac function, pattern and distribution of hypertrophy and tissue characterization without ionizing radiation [63, 64] thus representing an essential tool for the diagnosis and morphological assessment of HCM [64–67]. CMR allows the detection of unusual pattern of LV hypertrophy, such as lateral and apical distribution, which are not always easily visualized by echocardiography. Furthermore, CMR is a useful tool to evaluate the extent and severity of the hypertrophy in terms of mass quantification [5, 68] and to recognize right ventricular as well as papillary muscles hypertrophy, and mitral valve anomalies [69]. Moreover, CMR has also emerged as a valuable instrument to detect markers of the disease in patients with positive genotype but without LV hypertrophy (negative phenotype), such as myocardial crypts, elongated anterior mitral leaflet, abnormal apical trabeculae and smaller LV ventricular volumes [70] (Fig. 2).

CMR is helpful in the differential diagnosis between sarcomeric HCM and phenocopies or secondary hypertrophy, showing important differences in pattern and location of LV hypertrophy as well as in pattern and distribution of LGE and different values of native T1 [56, 64, 68, 71, 72]. CMR has also become an essential tool in the preoperative planning in patients undergoing septal reduction surgery [64, 73].

Areas of myocardial LGE representing replacement fibrosis [67, 68] are a common finding in this disease, expressed in up to 80% of HCM population [69], so that only quantitative analysis is a robust marker of unfavourable prognosis, in terms of progressive systolic dysfunction and malignant arrhythmias. A LGE threshold of 10–15% of LV mass have proved to be a possible cut off to identify patients at high risk of SCD, even in the absence of other major risk factors, who may benefit of primary prevention therapy [64 74–79]. Not surprisingly, the presence of LGE has been listed among the criteria to be considered in ICD patients selection in the recently updated HCM guidelines by AHA/ACC [64].

Also high signal intensity on T2-Weighted images has been demonstrated to predict arrhythmic events in the setting of HCM [80].

Although area of low ECV have been described in areas remote from hypertrophy, ECV is usually elevated in the hypertrophied areas both in patients with HCM as well as in phenotype-negative carriers of the disease [64, 81].

Diffusion Tensor (DT) CMR, visualizing microstructure of myocardial fibers, is an innovative sequence with the potential to represent myocardial disarray [82]. The latter technique, despite its complexity and limited availability, has the potentiality to provide further histopathological insights in the study of HCM and to offer additional markers of arrhythmic risk in HCM.

Finally, advanced analyses of standard technique might have clinical impact in the next future: a CMR Virtual Native Enhancement (VNE) can be generated from “cine” and native T1 mapping images using artificial intelligence, resembling conventional LGE without contrast administration [83]. Heterogeneity of scar, expressed as “dispersion map of LGE” may be a better marker of poor prognosis than its extent [84]. Another innovative post-processing analysis of LGE images enables to differentiate between the scar core and the border zone and to isolate corridors connecting the areas of normal myocardium to the scar core areas [85]. Lastly, we have to mention the role of bSSFP analysis in differentiating the different etiologies of HCM [86].

## Cardiac amyloidosis

Cardiac amyloidosis (CA) is a restrictive cardiomyopathy characterized by a pseudo-hypertrophy resulting by extracellular deposition of abnormal proteins in the myocardium [87]. Recently developed disease-modifying therapies increase the need of an early diagnosis [88]. Until recently, a positive biopsy was the only way to diagnose CA [88]. However, the combination of several imaging modalities has made possible a non-invasive diagnosis of CA, thus restricting the indication for EMB to those patients with equivocal or discordant clinical and imaging findings [88].

Although echocardiography remains the first line imaging modality in patients with suspected CA, CMR has shown to provide incremental information thanks to accurate morpho-functional evaluation, and tissue characterization [88]. Among recently published consensus documents, only one have proposed a “CMR” based pathway for the diagnosis of CA [89]. According to an ESC position paper, CMR can be used to implement the diagnostic algorithm of CA both in the “scintigraphy-based” and in the “laboratory-based” pathways, being particularly useful in patients with positive hematologic test and a negative scintigraphy (grade zero) [90].

To date, the key CMR technique to image CA is LGE, being the presence of diffuse subendocardial LGE highly specific for CA (94%). LGE imaging in patients with CA can be challenging in advanced stages due to the diffuse nature of LGE and to the equalization of myocardial and blood pool nulling point [22, 88]. However, the characteristic alterations in inversion times responsible of the aforementioned challenges in myocardial nulling, partially overcome by the development of phase sensitive inversion recovery (PSIR) sequences, are also strongly suggestive of the presence of amyloid deposits, supporting the diagnosis of CA [88, 93, 94].

Native T1 demonstrated high diagnostic accuracy in suspected CA with high positive and negative predictive values [95]. However, being T1 a composite signal from both the extra and intracellular space, it has turned out to be less specific than ECV, which to date represents the best parameter for quantifying amyloid and which has showed the best diagnostic accuracy when compared to other CMR parameters [96].

Beyond its role in the diagnostic workup of CA, CMR is important for prognostic information. The presence of LGE, especially when transmural, is a significant and independent predictor of mortality [88, 91, 97]. Furthermore, the aforementioned alterations in myocardial inversion times have also been found to be a negative prognostic marker thanks to their correlation with amyloid burden [22, 88, 92, 93].

ECV was found to be the parameter with the highest hazard ratio (as compared to LGE and native T1) in predicting patients’ prognosis, and its changes over the time could allow the assessment patients’ response to treatments [96, 98, 99]. The role of T2 mapping, adenosine stress perfusion and CMR-FT strain imaging have also showed to provide additional information in patients with CA, but further studies are needed to validate these findings in order to allow the application of these new techniques in daily clinical practice [100–105]. Supplementary material, case 2.

## Anderson fabry disease and other rare CMPs

Apart from sarcomeric HCM and amyloidosis, there are several other CMPs characterized by LV hypertrophy and therefore defined HCM mimics of phenocopies. Despite this overlapping phenotype, it is of extreme importance to correctly differentiate these entities, especially since specific treatments have become available to treat these conditions.

## Anderson fabry disease

Anderson-Fabry disease (AFD) is a rare X-linked inherited disorder caused by deficiency or absence of the enzyme α-galactosidase A (GLA), with subsequent accumulation of glycosphingolipids in several districts included the heart muscle cells and coronary circulation. The AFD clinical phenotype encompasses several scenarios due to the presence of different pathogenetic mutations in the GLA genes as well as to the X-linked inheritance of the disease, with homozygous males presenting with early signs and symptoms and heterozygous females experiencing milder phenotypes with later onset [106].

Although echocardiography remains the first line imaging examination in suspected AFD, CMR can help both in the differential diagnosis between AFD and sarcomeric HCM, as well as in the detection of subclinical stages of the disease. The main CMR findings in AFD are concentric LV hypertrophy [107] and non-ischemic mid-wall or subepicardial LGE pattern mainly involving the basal inferolateral LV segment [108]. In males, it seems that LGE does not precede the development of LV hypertrophy, while its presence has been reported in a significant proportion of female patients without hypertrophy [109]. The recently developed mapping techniques also provide useful data for the diagnosis of AFD. Indeed, intracellular accumulation of sphingolipids causes a typical shortening of native T1 relaxation times, even before the development of hypertrophy, and allows also to distinguish AFD from other hypertrophic diseases, typically characterized by elevated T1 values [109, 110]. However, it is also important to remember that during the disease course, the development of myocardial fibrosis, secondary to myocardial inflammation mediated by sphingolipid, balances the effect of sphingolipid on T1 relaxation times leading to a pseudo-normalization of native T1, at least in myocardial regions involved by fibrosis. Among parameters derived from mapping analysis, ECV is typically normal in AFD because of the intracellular accumulation of sphingolipids, as compared to other CMPs characterized by interstitial infiltration (e.g., amyloidosis). In fact, ECV values reflect the increase of the extracellular space, typically not affected in AFD [56]. Finally, T2 mapping has been used to demonstrate the presence of myocardial inflammation, which is thought to contribute to disease progression [111–113].

Recently, both enzyme replacement therapy (ERT) and chaperone therapy have demonstrated to be safe and effective in stabilizing the disease course and improving symptoms in patients affected by AFD. The initiation of ERT treatment is yet recommend for patients exhibiting symptoms and LV hypertrophy. CMR techniques hold strong potential in AFD not only for guiding the appropriate timing for ERT introduction and prognostic classification, but also for monitoring response to therapy. For instance, several studies reported more effective results of ERT in terms of LV mass regression when little or no LGE was present at baseline evaluation [114] thus suggesting that specific treatment should be initiated earlier, as soon as the first structural or functional cardiac abnormalities become detectable and before development of myocardial fibrosis. Supplementary, material case 3.

## Cardiac siderosis

Iron overload cardiomyopathy can occur in patients affected by genetic haemochromatosis or, more commonly, it can be secondary to excessive iron administration in subjects requiring repeated blood transfusion as it happens in the setting of hereditary anemias. When left untreated, it can lead to heart failure and even death. After the introduction of mapping techniques, CMR has become an essential tool in the diagnosis and risk stratification of this condition. In fact, the myocardial iron deposits affect T2* relaxation time, thus allowing the diagnosis of cardiac siderosis. Furthermore, a linear relationship between the reduction in T2* and the amount of iron in myocardium and an increased risk of ventricular arrhythmias has been demonstrated. Therefore, to date different cut offs of T2* are used to diagnose iron overload CMP and to guide the initiation of iron chelation therapy, as well as to monitor patients’ response to medical treatment, with a dramatic improvement in the prognosis of these patients [115]. Native T1 is also decreased in 10 and can be used for diagnosis [116].

## Glycogen storage disease

Glycogen storage diseases (e.g., Pompe, PRKAG2, Danon) may determine severe increase in LV mass with rapid progression toward heart failure. CMR may be helpful also in the assessment of these rare CMP, for instance Danon disease is characterized by extensive LV subendocardial LGE, particularly at apical level, with sparing of basal septum [117]. However, because of the scarce amount of data, the role of CMR in determining prognosis in these rare conditions still needs to be defined.

## LV noncompaction—anatomical phenotype or a distinct entity?

LV noncompaction (LVNC) is a heterogeneous entity characterized by the presence of extensive myocardial trabeculations and currently listed among “not classified CMPs.” Traditionally, the presence of this characteristic ventricular pattern has been attributed to the arrest of normal embryogenesis of the endocardium and myocardium or to an abnormal myocardial development, which recognize a genetic background in one third of cases, with mutation in genes encoding for sarcomeric and cytoskeletal proteins being the most represented [1, 118, 119]. Furthermore, several genetic mutations have been associated with the presence of LV systolic dysfunction and a more severe prognosis [120]. Despite those proved genetic determinants, there are growing data demonstrating the presence of reversible forms of LVNC related to overload conditions (i.e., strenuous training, pregnancy), thus suggesting that LVNC should be considered as an anatomical phenotype rather than a real CMP [119]. The definition of this entity in clinical practice has always been challenging especially due to an overlap with other cardiomyopathies and with normal LV trabeculation [22]. CMR has become a valuable tool for the non-invasive assessment of patients with a suspected LVNC. Several diagnostic criteria have been proposed, among these the two most widely used are those proposed by Petersen and Jacquier which require the presence of a NC to C ratio of 2.3/1 and the detection of a trabeculated LV mass > 20% of the LV global mass, respectively [22, 121, 122]. All these proposed CMR diagnostic criteria have showed to be highly sensitive but also non-specific, with several normal individuals meeting at least one criterion for LVNC according to a recent study [123]. Furthermore, in asymptomatic subjects the presence of LVNC as diagnosed by the aforementioned CMR criteria have showed no progression at 10 years follow up [124]. Similarly, 1,4% of athletes meet the diagnostic criteria for LVNC at CMR but only a small percentage of them (0,1%) have also LV dysfunction or a positive family history. Therefore, since it has been demonstrated that in absence of symptoms, positive family history, left ventricular systolic dysfunction or LGE, the event-rate during follow up is very low [125], CMR criteria should be integrated with clinical data in order to improve the specificity of LVNC diagnosis [86]. Recently, an individualized model for prognostic risk stratification has been proposed. This model, which considers also the presence of LGE on CMR, is based on a multicenter retrospective study enrolling 585 patients and showing that LVNC was associated with a higher risk of adverse outcome during follow-up in the presence of LV systolic dysfunction or in patients with preserved LVEF but with LGE at CMR [126].

At the same time, additional CMR markers could be validated in the future to discriminate individuals with an increased risk of events at follow up, among these the presence of LV systolic dysfunction and LGE has already demonstrated to correlate with a worse prognosis especially when associated with LV dysfunction [125, 127, 128].

Table 2 resumes the main diagnostic and prognostic CMR findings for both dilated and hypertrophic phenotype.

**Table 2:** Main diagnostic and prognostic CMR findings in CMPs with dilatative and hypertrophic phenotype

	**Dilatative phenotipe**			**Hypertrophic phenotipe**		
	**DCM**	**ACM**	**Myocarditis**	**HCM**	**AFD**	**Amyloidosis**
**Diagnosis**	**Cine imaging**: reduced left/biventricular systolic function, possible left/bi ventricular dilatation ***T2 weighted imaging****: differential diagnosis from “acute inflammatory” cardiomyopathies* **LGE**: in up to 30–40% of cases, typically midwall pattern in the interventricular septum. Other patterns are possible (subepicardial pattern in post inflammatory DCM) **Mapping**: altered T1 and ECV mapping reflecting the presence of interstitial fibrosis ***Feature tracking analysi****s: may be more accurate in the detection of impaired contractility*	**Cine with additional sequences for right ventricle**: RV or biventricular morpho-functional abnormalities Detection of fatty infiltration from “India-Ink” artifact ***T2 weighted imaging:**** in case of “hot-phases” presentation; differential diagnosis from “acute inflammatory” cardiomyopathies* **FSE**: fatty infiltration of the RV and/or LV (might be replaced by the assessment of “India Ink” artifact from SSFP) **LGE**: detection of areas of fibro fatty myocardial replacement. Commonly in the subepicardial layers of the LV free wall, expecially in the inferolateral region, with or without septal involvement. Possible LGE “ring pattern” in some left dominant variants **Mapping**: still limited applications ***Feature tracking analysis****: could allow the detection of segmental impairment of wall contraction in early phases*	**Cine imaging**: detection of ventricular systolic dysfunction, and impaired contractility Detection of hyperemia when acquired immediately after Gd injection **T2 weighted imaging**: detection of edema in the acute phase **LGE**: detection of necrosis (acute phase)/ fibrosis (healed myocarditis) **Mapping**: increased T1 mapping/ECV values in both acute and chronic forms, altered T2 mapping values in the acute phase ***Feature tracking analysi****s: may be more accurate in the detection of impaired contractility*	**Cine imaging**: assessment of ventricular mass, chamber volume, cardiac function, pattern and distribution of hypertrophy. Detection of right ventricular as well as papillary muscles hypertrophy In genotype positive phenotype negative subjects: detection of myocardial crypts, elongated anterior mitral leaflet, abnormal apical trabeculae and smaller LV ventricular volumes **Special sequencese for the evaluation of LVOT obstruction** ***T2 weighted imaging:**** detection of oedema/ongoing interstitial remodelling* **LGE**: found in up to 80% of HCM population **Mapping**: altered T1 and ECV mapping reflecting the presence of interstitial fibrosis	**Cine imaging**: assessment of ventricular mass, chamber volume, cardiac function, pattern and distribution of hypertrophy (Typically concentric) **LGE**: typically with non-ischemic mid wall or subepicardial pattern, mainly involving the basal, inferolateral LV segment **Mapping**: reduction in native T1 in the early phase of the disease with following pseudo normalization. Normal ECV values	**Cine imaging**: assessment of ventricular mass, chamber volume, cardiac function **LGE**: diffuse subendocardial LGE (highly specific for CA). Difficult myocardial nulling ***TI scout:**** useful to detect a very short myocardial nulling time (before or close to the bloodpool), typical of advanced forms of cardiac amyloidosis* **Mapping**: high T1 values for non-contrast diagnosis; high ECV values, parameter with best diagnostic accuracy
**Prognosis**	**Cine imaging**: detection and assessment of right ventricular dysfunction **LGE**: predictor of adversed prognosis. The impact of LGE extension as well as the role of the different LGE locations are still debated **Feature tracking analysis**: reduced GLS was could correlate with a worse outcome, still needs validation **Mapping**: altered T1 and ECV mapping predictors of adverse prognosis in small studies	**Cine imaging**: assessment of biventricular dysfunction **LGE**: the prognostic impact of LGE extension and location are still debated	**T2 weighted imaging**: abnormal findings correlate with a worse outcome **LGE**: negative prognostic value especially if located in the interventricular septum **Feature tracking analysis**: reduced GLS may correlate with a worse outcome, still under evaluation	**LGE**: the presence of a total LGE of more than 10–15% of LV mass correlates with increased risk of SCD **Mapping / dimensions phase contrast CMR (4D flow/ dispersion map of LGE/ Contrast-enhanced CMR**: under evaluation		**LGE**: independent predictor of mortality, especially when transmural **Mapping**: ECV values are the best parameter to predict patients’ prognosis

In *Italics* are highlighted the optional techniques

## Conclusion

Today, more than ever before, a patient-tailored approach is mandatory in every medical field, and particularly in CMPs. In fact, the growing body of knowledge on patho-physiological pathways, diagnostic and prognostic work-up of CMPs as well as the availability of an increasing number of targeted disease-modifying therapies make it mandatory to achieve a timely diagnosis and a precise characterization of the different phenotypes of CMP.

Recent advances in CMR and its increased accessibility allow a precise assessment of ventricular dimension and function as well as a non-invasive tissue characterization of the myocardium. However, the growing knowledge deriving from CMR studies should always be interpreted in light of clinical elements and integrated with information derived by other imaging techniques (Table 3), such as echocardiography (which remains the first line imaging tool to guide the diagnosis in patients with suspected CMP) and genotype or histological information. CMR, thanks to its ability to add information about tissue characterization, appears to be particularly relevant in subclinical and recently onset CMPs, as well as in genotype positive phenotype negative subjects [129]. New imaging techniques both for echo and for CMR (i.e. diffusion tension imaging, speckle and feature tracking and myocardial work, T1/T2 mapping) are increasingly used in experienced labs to help clinicians in the differential diagnosis and management of specific CMP subtypes (i.e. Amyloid or Anderson Fabry disease) [129]. Although the increased enthusiasm for the use of CMR in the diagnosis and characterization of CMP, it has to be recognized that a multimodality imaging approach remains the gold-standard, mostly for challenging settings such as infiltrative cardiomyopathies [129, 130].

**Table 3:** Comparison of different cardiovascular imaging modalities for the management of cardiomyopathies

	**TTE**	**CMR**	**SPECT/PET**	**CT**
**Cardiac morphology and function**				
Chamber volumes	+ +	+ + +	+	+ +
Wall thickness	+ +	+ + +	-	+ +
Systolic function	+ +	+ + +	+	+
Diastolic function	+ + +	+ +	+	+
Myocardial mechanics	+ +	+ + +	-	+
**Myocardial tissue characterization**				
Fibrosis	+	+ + +	+	+ + (CT-DE)
Inflammation	-	+ + +	+ + + (FDG-PET)	+
Amyloidosis	+	+ + +	+ + +	-
Ischaemia/CAD	+ + (stress)	+ + + (stress)	+ + + (stress)	+ + + (CCTA/stress)
Myocardial metabolism	-	+ + (MRS)	+ + +	-
Myocardial innervation	-	-	_+++_(MIBG)	-
**Valvular assessment**				
Valve morphology	+ + +	+ +	- + +	
Cardiac haemodynamics	** + + + **	** + **	**–**	
Valvular stenosis	+ + +	+	- +	
Valvular regurgitation	+ + +	+ +	- +	
**Pericardial assessment** Effusion/tamponade	+ + +	+ + +	-	+ +
Inflammation	-	+ + +	+ + (FDG-PET)	+ +
Constriction	+	+ + +	-	+ +
**Technical characteristics** Availability	+ + +	+	+	+ +
Fast acquisition	+ + +	-	-	+ +
Spatial resolution (mm)	0.5–2	1–2	4–8(PET)/5–15(SPECT)	0.5
Temporal resolution (ms)	< 10	20–50	100–300	80–135
Feasibility in patients with severe renal failure	+ + +	+	+ + +	-
arrhythmias	+ + +	+	+ +	+
pacemaker/defibrillators	+ + +	+	+ + +	+ +
claustrophobia	+ + +	+	+ +	+ +
obesity	+	+ +	+ +	+ + +
COPD	+	+ + +	+ + +	+ + +
pregnancy	+ + +	+ +	-	-

*CAD*, coronary artery disease; *CCTA*, coronary computed tomography angiography; *CMR*, cardiac magnetic resonance; *COPD*, chronic obstructive pulmonary disease; *CT*, computed tomography; *CT-DE*, delayed enhancement at CT; *FDG*, fluoro-deoxy-glucose; *MIBG*, meta-iodo-benzyl-guanidine; *MRS*, magnetic resonance spectroscopy; *PET*, positron emission tomography; *SPECT*, single-photon emission computed tomography; *TTE*, transthoracic echocardiography

In conclusion, an integrated clinical and imaging approach seems to be essential to guide diagnosis, define the different CMP phenotypes (HCM, DCM, arrhythmogenic cardiomyopathy, restricted cardiomyopathy, LVNC) and unravel specific underlying aetiologies as well as to ensure a tailored therapeutic management and predict disease prognosis.

## Supplementary Information

Below is the link to the electronic supplementary material.

Supplementary file1 (DOCX 48019 KB)
