# Supplementary material for: Clinical application of CMR in cardiomyopathies: evolving concepts and techniques: A position paper of myocardial and pericardial diseases and cardiac magnetic resonance working groups of Italian society of cardiology
Source: Heart Fail Rev. 2022 May 10;28(1):77–95. doi: 10.1007/s10741-022-10235-9 (PMC9902331; doi:10.1007/s10741-022-10235-9)
Supplement: Supplementary file 1 — Supplementary file1 (DOCX 48019 KB) [file 10741_2022_10235_MOESM1_ESM.docx]

**Supplemental table 1. Overview of experimental CMR sequences and AI applications in CMR**

| Name | Description | Ref |
| --- | --- | --- |
| **Novel experimental CMR sequences** | | |
| Diffusion tensor CMR | Detects helical cardiomyocyte arrangement that drives rotation and torsion; abnormal myocyte orientation and sheetlet function has been demonstrated in congenital heart disease, ischemic and nonischemic cardiomyopathies | 1 |
| Oxygenation-sensitive CMR | Detects myocardial oxygen delivery/extraction, based on the ratio of deoxyhemoglobin (paramagnetic) over oxyhemoglobin (diamagnetic) in T2*-weighted blood-oxygen level-dependent (BOLD) sequences; in ischemic myocardium, oxygen extraction increases more than blood supply, causing a higher deoxyhemoglobin concentration and a low MR signal | 2, 3 |
| Hyperpolarized CMR | Assessment of cardiac metabolism, based on the hyperpolarization of physiological molecules as contrast agents; hyperpolarized [1-13C] pyruvate looks particularly promising for the assessment of cardiac energy metabolism | 4 |
| Magnetic resonance elastography | Detection of myocardial stiffness, using an external vibrating source that generates shear waves inside a tissue of interest, then vibrational displacements are measured in the tissue and a stiffness map is generated | 5 |
| **Novel approached to CMR data acquisition and analysis** | | |
| Artificial intelligence | Automated cardiac segmentation and contouring for image post-processing analysis  Extraction of voxel-level information from conventional CMR images and conversion into multiple numeric data (features)  Fast image reconstruction algorithm using all acquired raw data: while established techniques such as compressed sensing and parallel imaging are based on k-space undersampling and need an explicitly defined reconstruction algorithm, deep neural networks exploit large data sets to learn the key reconstruction parameters and to provide 2D or 3D images from all acquired data | 6  7  8 |

*1 Z. Khalique, P.F. Ferreira, A.D. Scott, S. Nielles-Vallespin, D.N. Firmin, D.J. Pennell, Diffusion Tensor Cardiovascular Magnetic Resonance Imaging: A Clinical Perspective, JACC Cardiovasc. Imaging. (2019). doi:10.1016/j.jcmg.2019.07.016*

*2 M.G. Friedrich, T.D. Karamitsos, Oxygenation-sensitive cardiovascular magnetic resonance., J. Cardiovasc. Magn. Reson. 15 (2013) 43. doi:10.1186/1532-429X-15-43*

*3* *Chen BH, Wu R, An DA, Shi RY, Yao QY, Lu Q, Hu J, Jiang M, Deen J, Chandra A, Xu JR, Wu LM. Oxygenation-Sensitive Cardiovascular Magnetic Resonance in Hypertensive Heart Disease With Left Ventricular Myocardial Hypertrophy and Non-Left Ventricular Myocardial Hypertrophy: Insight From Altered Mechanics and Cardiac BOLD Imaging. J Magn Reson Imaging. 2021 Mar;53(3):965-966. doi: 10.1002/jmri.27401. Epub 2020 Oct 17. Erratum for: J Magn Reson Imaging. 2018 Nov;48(5):1297-1306. PMID: 33068066.*

*4. A.J.M. Lewis, D.J. Tyler, O. Rider, Clinical Cardiovascular Applications of Hyperpolarized Magnetic Resonance, Cardiovasc. Drugs Ther. (2020). doi:10.1007/s10557-020-06942-w*

*5 A. Arani, S.P. Arunachalam, I.C.Y. Chang, F. Baffour, P.J. Rossman, K.J. Glaser, J.D. Trzasko, K.P. McGee, A. Manduca, M. Grogan, A. Dispenzieri, R.L. Ehman, P.A. Araoz, Cardiac MR elastography for quantitative assessment of elevated myocardial stiffness in cardiac amyloidosis, J. Magn. Reson. Imaging. 46 (2017) 1361–1367. doi:10.1002/JMRI.25678*

*6 N. Goyal, V. Mor-Avi, V. Volpato, A. Narang, S. Wang, M. Salerno, R.M. Lang, A.R. Patel, Machine learning based quantification of ejection and filling parameters by fully automated dynamic measurement of left ventricular volumes from cardiac magnetic resonance images, Magn. Reson. Imaging. 67 (2020) 28–32. doi:10.1016/j.mri.2019.12.004*

*7 Raisi-Estabragh, C. Izquierdo, V.M. Campello, C. Martin-Isla, A. Jaggi, N.C. Harvey, K. Lekadir, S.E. Petersen, Cardiac magnetic resonance radiomics: basic principles and clinical perspectives., Eur. Heart J. Cardiovasc. Imaging. 21 (2020) 349–356. doi:10.1093/ehjci/jeaa028*

*8 A. Bustin, N. Fuin, R.M. Botnar, C. Prieto, From Compressed-Sensing to Artificial Intelligence-Based Cardiac MRI Reconstruction, Front. Cardiovasc. Med. 7 (2020) 17. doi:10.3389/fcvm.2020.00017*

**Clinical cases**

**Case 1**

A 75-year-old man was referred to the dedicated arterial hypertension outpatient clinic for cardiovascular risk assessment by his general practitioner. Medical history included arterial hypertension, active cigarette smoking, carotid atheromasia, impaired fasting glucose, overweight. He was already on aspirin, olmesartan, hydrochlorothiazide, bisoprolol.

The patient was asymptomatic for chest pain, dyspnea, palpitations. He reported no history of syncope. Cardiopulmonary clinical examination was unremarkable. 12-leads electrocardiogram (ECG) was performed, showing sinus rhythm, 65bpm, normal atrio-ventricular conduction, interventricular conduction delay (QRS 110msec), low voltages in the peripheral leads, fractioned QRS in V1-V2, DIII and aVF leads, QTc 430msec (Figure 1).

The patient was referred for transthoracic echocardiography. Image quality was poor due to an inadequate acoustic window. The left ventricle appeared mildly dilated, with regional wall motion abnormalities and mildly reduced ejection fraction. Bi-atrial dilatation and mild pericardial effusion were also noted. Other findings were normal.

The patient was referred for cardiac magnetic resonance (CMR) for a more in-depth assessment of morphology and function and for tissue characterization. Considering the high cardiovascular risk and regional wall motion abnormalities, adenosine first-pass perfusion assessment was also requested.

CMR showed high-normal left ventricle (LV) end-diastolic volume (EDV) (195 ml [105-187ml]; 92ml/m^2^ [58-93 ml/m^2^]), hypokinesia of the inferior, posterior and lateral walls with mildly reduced ejection fraction (EF) (LVEF 51% [59-77%]). Right ventricle (RV) was dilated (RVEDV 210ml [100-200 ml]; 99ml/m^2^ [52-98ml/m^2^]) with hypokinesia of the free wall and reduced EF (RVEF 52% [57-83%]). Extensive areas of subepicardial and mid-ventricular fibro-fatty infiltration were observed in both ventricles at T1-weighted and Dixon-separated fat-water images (1) and at late gadolinium enhancement imaging (Figures 2, 3 and 4).

At in-line first-pass perfusion mapping (1) inducible perfusion defect was observed in the septum, inferior, posterior and lateral walls (Figure 5).

The diagnosis of biventricular arrhythmogenic cardiomyopathy (AC) was made accordingly to the Padua criteria (≥1 morpho-functional and/or structural abnormalities of both the RV and LV) (2) The patient was then referred to the Cardiomyopathy Clinic, where he is undergoing additional testing (coronary CT, 24-hour Holter ECG, exercise test, genetic testing).

This case illustrates the incremental value of CMR in the diagnosis of AC. The patient did not report cardiac symptoms, but the ECG was abnormal. Echocardiography, limited by an inadequate acoustic window, was suspicious for LV dilatation and dysfunction, with regional wall motion abnormalities that could have been interpreted as of ischemic origin. CMR not only confirmed the LV findings, but also demonstrated RV dilatation and dysfunction, and extensive fibro-fatty infiltration. The finding of reduced peak stress perfusion was consistent with areas of fibro-fatty replacement.

Although this patient is 75 years old and has always been asymptomatic, this diagnosis can impact family members.


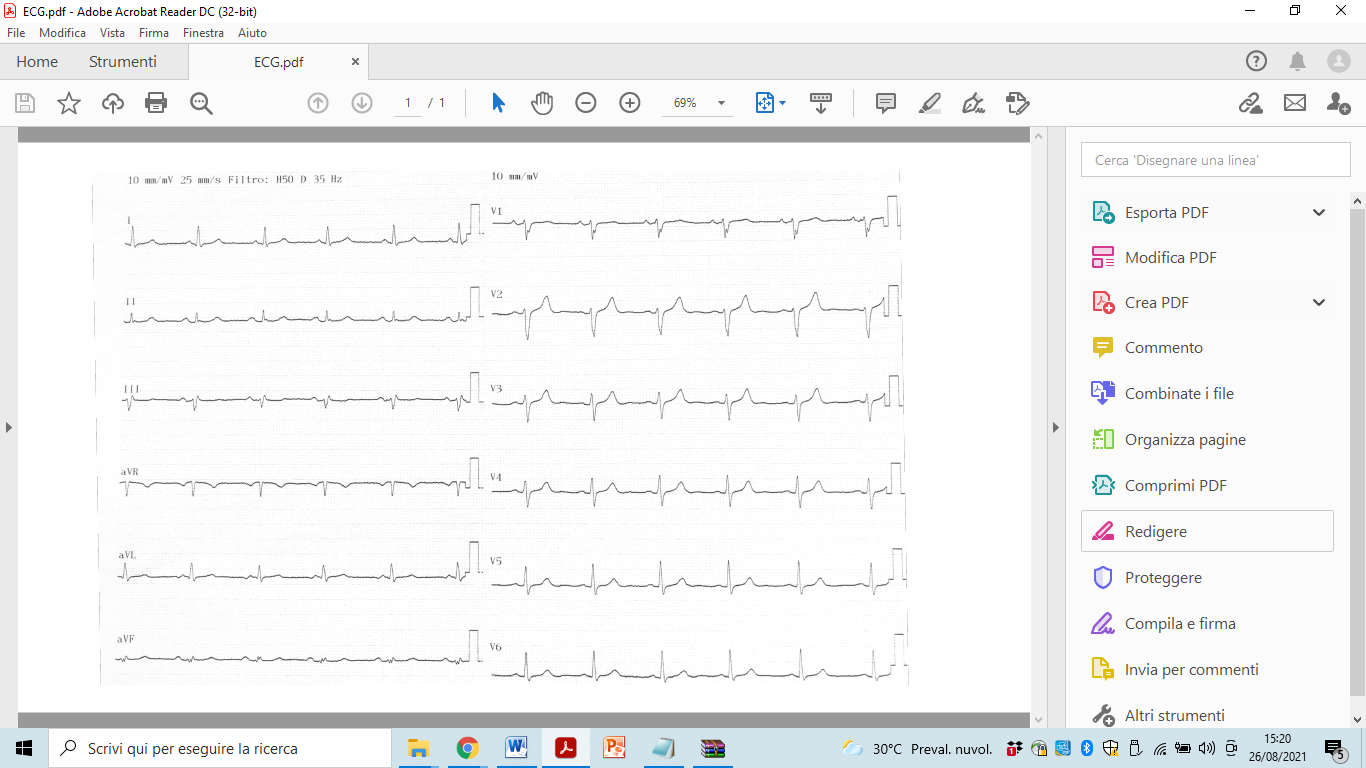


Figure 1: 12-leads ECG

Figure 2: 4-, 2- and 3-chambers view. Panel A: b-SSFP cine. Subepicardial and mid-wall fatty infiltrations are already visible as hyperintense areas inside the myocardium in the lateral mid-apical wall, anterior distal wall, apical septum and posterior wall (red arrows). Panel B: T1-weighted images. Fat appears hyperintense (red arrows) and can also be observed in right ventricle free wall.

Figure 3: Dixon-separated fat water 4-, 2- and 3-chambers views. Panel A: fat. Panel B. water. Red arrows indicate fatty infiltration, hyperintense in the fat image and suppressed in the water image. Subepicardial and mid-wall fatty are visible in the lateral mid-apical wall, anterior distal wall, apical septum and posterior wall. Fat can also be observed in right ventricle free wall (red arrows).

Figure 4: Motion-corrected segmented inversion-recovery and phase-sensitive-inversion-recovery gradient-echo sequence late-gadolinium enhancement (LGE) imaging (3) Panel A: 4-, 2- and 3-chambers views. Panel B: basal, mid-ventricular and apical short axis. Red arrows indicate LGE. Subepicardial and mid-wall fatty LGE is visible in the lateral mid-apical wall, anterior distal wall, apical septum and posterior wall. LGE can also be observed in right ventricle free wall.

Figure 5: In-line quantitative perfusion mapping (1) acquired at peak adenosine stress (Panel A) and at rest (Panel B) in basal, mid-ventricular and apical short axis view (from left to right). An inducible perfusion defect may be observed in the basal posterior septum, inferior, posterior and lateral walls; in the mid ventricular posterior septum and inferior wall; a thin subendocardial rim in the apical septum and inferior wall.

1 *Kellman P, Hansen MS, Nielles-Vallespin S, Nickander J, Themudo R, Ugander M, Xue H. Myocardial perfusion cardiovascular magnetic resonance: optimized dual sequence and reconstruction for quantification. Journal of Cardiovascular Magnetic Resonance. 2017;19. doi:10.1186/s12968-017-0355-5.*

*2 Corrado IJC 2020 doi: 10.1016/j.ijcard.2020.06.005*

*3 Motion corrected free-breathing delayed-enhancement imaging of myocardial infarction using nonrigid registration.*

*Ledesma-Carbayo MJ, Kellman P, Hsu LY, Arai AE, McVeigh ER*

*J Magn Reson Imaging. 2007 Jul; 26(1):184-90.*

**Case 2**

A 70 year-old was admitted to our Neurological Science Department for recurrent dizziness associated with palpitations.

He had already undergone exercise testing, carotid artery echography, 24-HolterECG monitoring and transthoracic echocardiogram (TTE) that had been reported normal, except for some degree of septal cardiac hypertrophy.

His medical history included arterial hypertension; diabetes; dyslipidemia; history of spinal disc herniation corrected by surgery.

He was already on nebivolol, spironolactone and simvastatin.

During admission he underwent a repeated 24-HolterECG monitoring that showed I-degree atrioventricular block, short SVT runs and two NSVT.

TTE confirmed cardiac hypertrophy and therefore he underwent cardiac magnetic resonance (CMR).

CMR showed normal LV size (EDV 134 ml [105-187ml]; 75 ml/m^2^ [58-93 ml/m^2^], ESV 47 ml [25-70 ml]; 26 ml/m^2^ [13-35 ml/m^2^]), concentric LV hypertrophy (MWT 20 mm in the mid septum) and increased LV mass (189 g [106-183 g]; 105 g/m2 [56-89 g/m2]) and hypertrophied papillary muscles; normal RV size and function (RVEDV 103ml [100-200 ml]; 57ml/m^2^ [52-98ml/m^2^]; RVEF 83% [57-83]) and RV hypertrophy (MWT 9 mm in the basal free wall; biatrial dilatation (LAA 17 cm2/m2, RAA 15 cm2/m2) (figure 1). Native T1 value was high (1290 ±50 msec, normal values on scanner 920-1040msec) with extensive oedema noted at T2 mapping images. Also extracellular volume (ECV) value was high: 48% (figure 2). After injection of contrast, alterated kinetic was noted. There was soft late gadolinium enhancement in all the basal segments, almost transmural in the septum; papillary muscles were heavily affected whilst there was a partial apical sparing (figure 3).

The suspicion of cardiac amyloidosis was raised and the patient underwent total body PET that demonstrated findings consistent with cardiac amyloidosis.

Genetic testing was performed and no mutations were found.

Therefore the diagnosis of TTR-wt amyloidosis was performed.

As he was also symptomatic for dyspnoea, he underwent cardiopulmonary exercise testing and six-minute walking test that showed signs of marked heart failure. Therefore he was started on tafadimis.

This case illustrates the incremental value of CMR in performing the correct diagnosis, even beyond the genetic testing. Correct diagnosis is important also to start a targeted therapy.


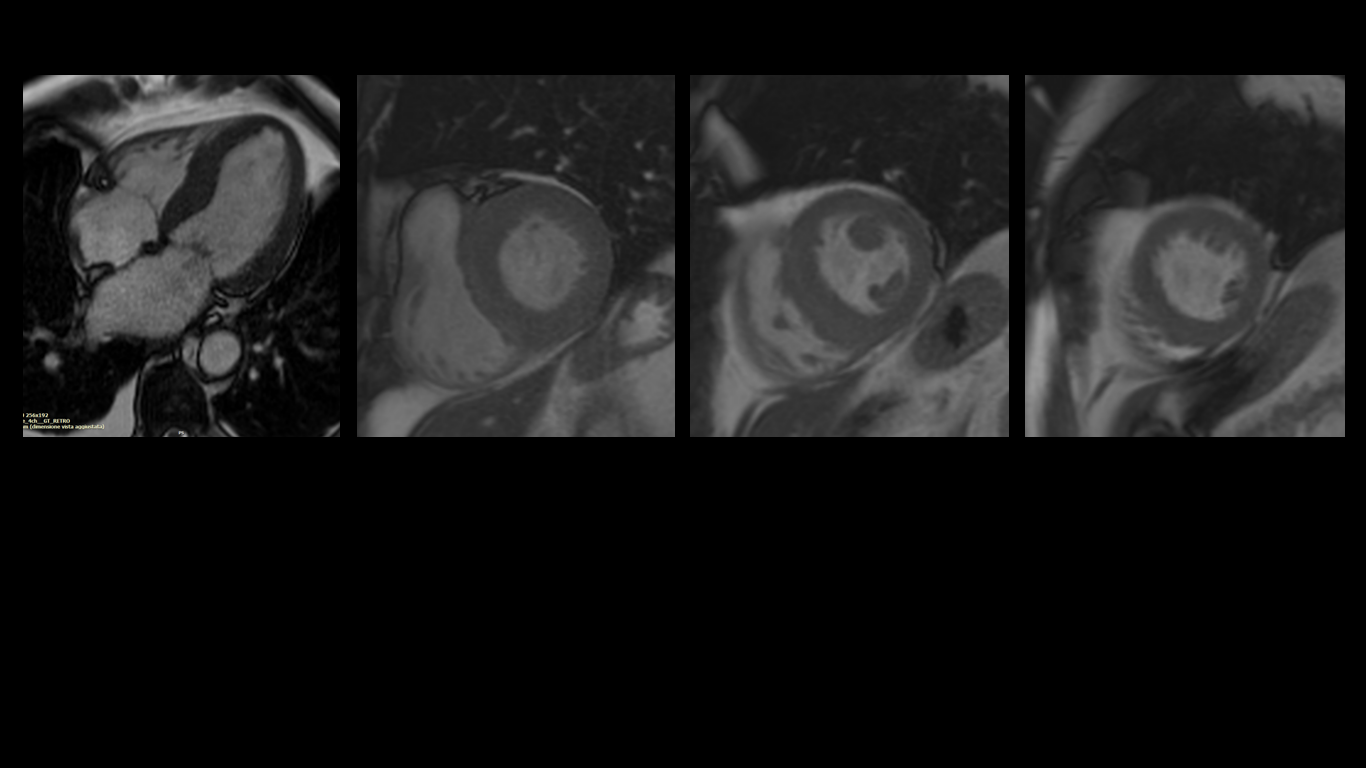


Figure 1: from left to right, SSFP-cine 4-chambers and basal, mid-ventricular and apical short axis views.


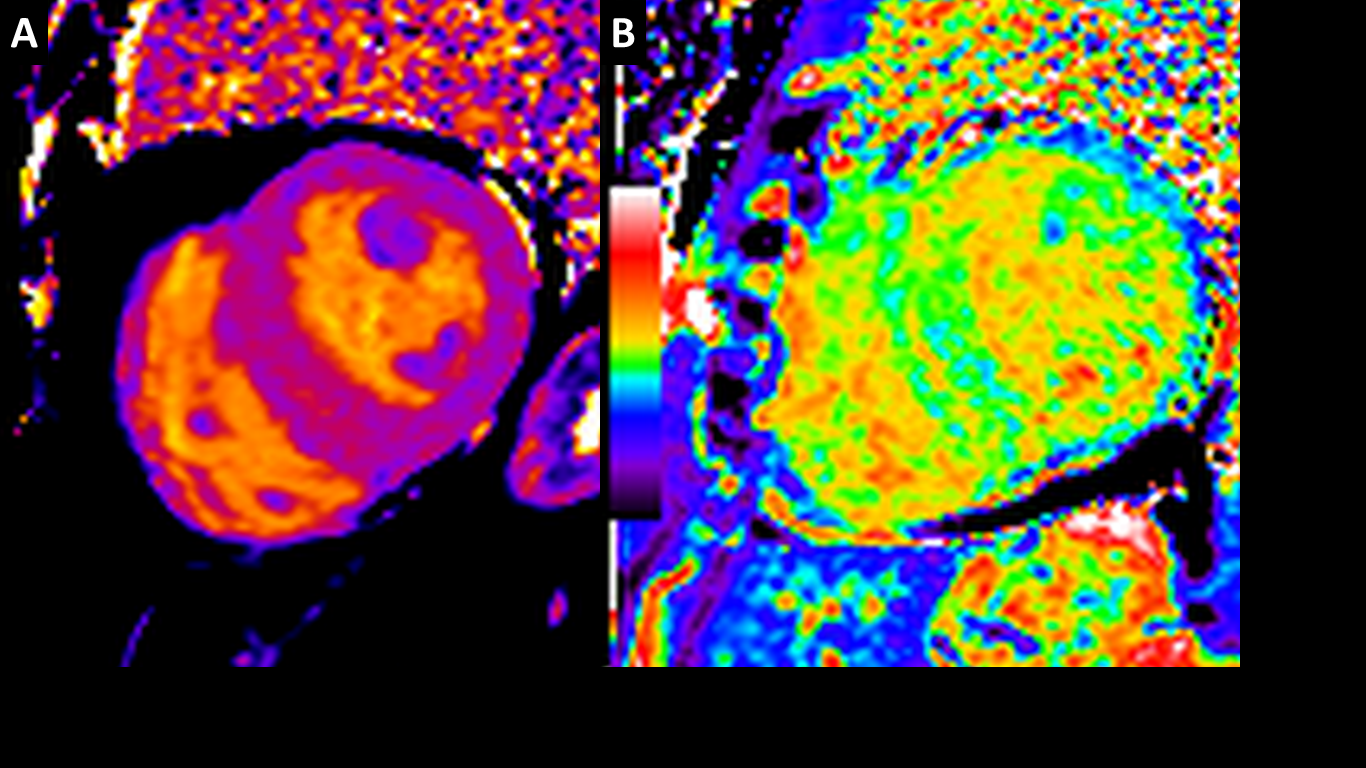


Figure 2: Panel A: native mid-ventricula T1 mapping (Modified look-locker inversion recovery, MOLLI) is increased in the septum. Panel B: synthetic extracellular volume (ECV) map show severely increased ECV: 48%.

**Case 3**

A 63y.o. gentleman was referred to the Cardiomyopathy clinic after deep inverted T waves had been noted on a 12-leads electrocardiogram (ECG) performed during a routine cardiological assessment for hypertension.

His medical history included arterial hypertension since age 45; left-sided hypoacusia; deep vein thrombosis aged 50; impaired kidney function since age 50 and kidney transplantation aged 62 due to undetermined glomerulosclerosis. He was on therapy with carvedilol, amlodipine, aspirin, omeprazole and cortisone.

His brother had history of proteinuria and seizures, no sudden death had occurred in the family. The patient reported no symptoms. On physical examination a mild systolic murmur was noted. The ECG confirmed the presence of deep T waves inversion in the infero-lateral leads (figure 1).

The echocardiogram showed normal biventricular size and function, concentric left ventricle (LV) hypertrophy with 20 mm of maximal wall thickness (MWT), hyperechogenic postero-lateral and septal walls, upper normal right ventricle (RV) wall thickness (5 mm), mild left atrial (LA) dilatation and mild mitral regurgitation.

Overall, the clinical suspicious was of Anderson-Fabry disease with multiorgan involvement, included cardiac hypertrophy.

Blood sample for genetic testing was taken and the patient referred for cardiac magnetic resonance (CMR) for a better morphological assessment and for tissue characterization.

CMR showed normal LV size (EDV 179 ml [109-191ml]; 93 ml/m^2^ [60-95 ml/m^2^], ESV 42 ml [27-72 ml]; 22 ml/m^2^ [14-36 ml/m^2^]), concentric LV hypertrophy (MWT 23 mm in the mid posterior septum) and markedly increased LV mass (347 g [107-183 g]; 180 g/m2 [57-90 g/m2]), a small LV crypt and multiple papillary muscles; normal RV size and function and concentric RV hypertrophy (MWT 9 mm in the mid free wall); mild dilated LA (19 cm2/m2). Native T1 value normal due to the presence of extensive oedema was noted at T2-weighted and T2 mapping images, mainly in the lateral wall and in all the distal segments. There was late gadolinium enhancement in the posterior-lateral wall, in the mid-apical anterior wall and soft patchy late enhancement in the remaining segments (figure 2).

The findings confirmed the clinical suspicious of Anderson-Fabry disease. The genetic testing resulted positive c.644A>G disease-causing mutation on GLA.

This case illustrate the incremental value of CMR in the morphological definition: CMR was able to better define the degree of hypertrophy both of the LV and RV. It also allowed a detailed tissue characterization that confirmed the diagnosis even before the genetic testing result. The right diagnosis is crucial also for targeted therapy.


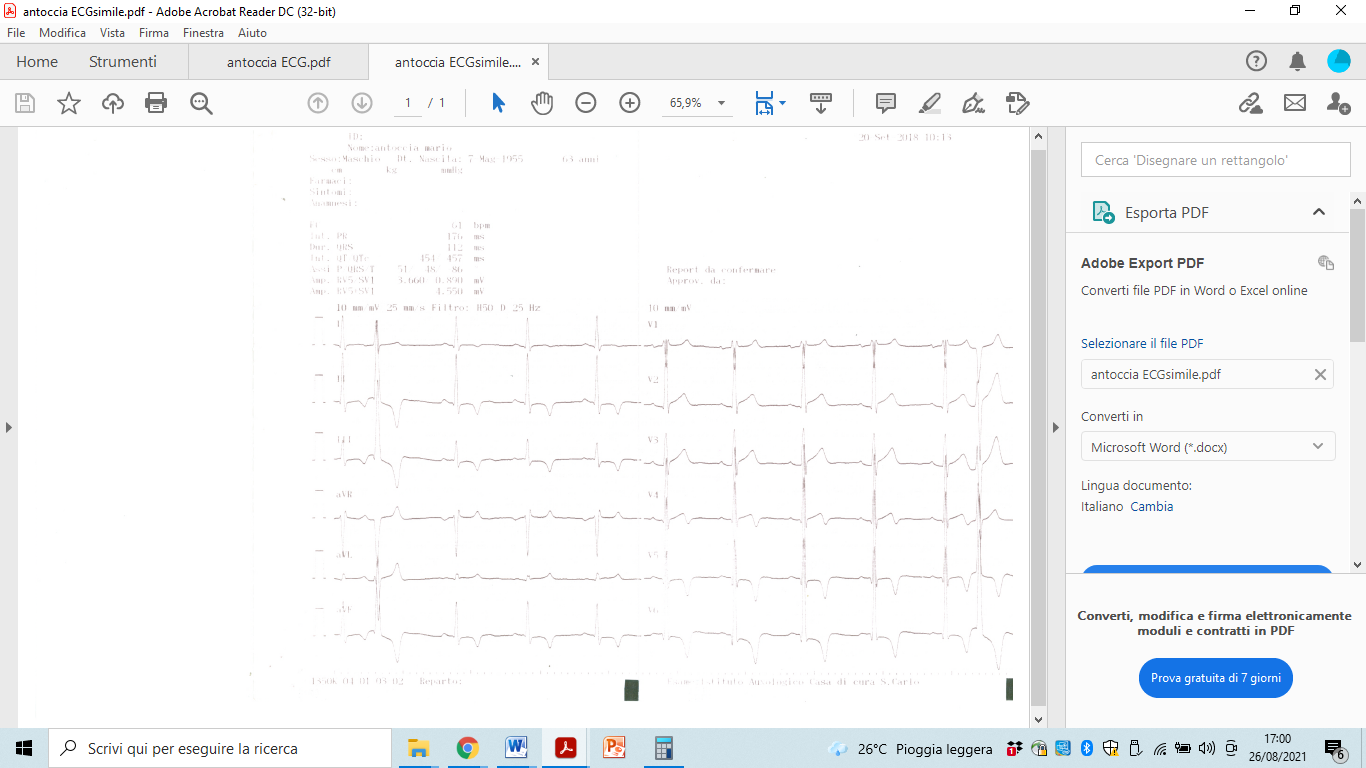


Figure 1: 12-leads ECG


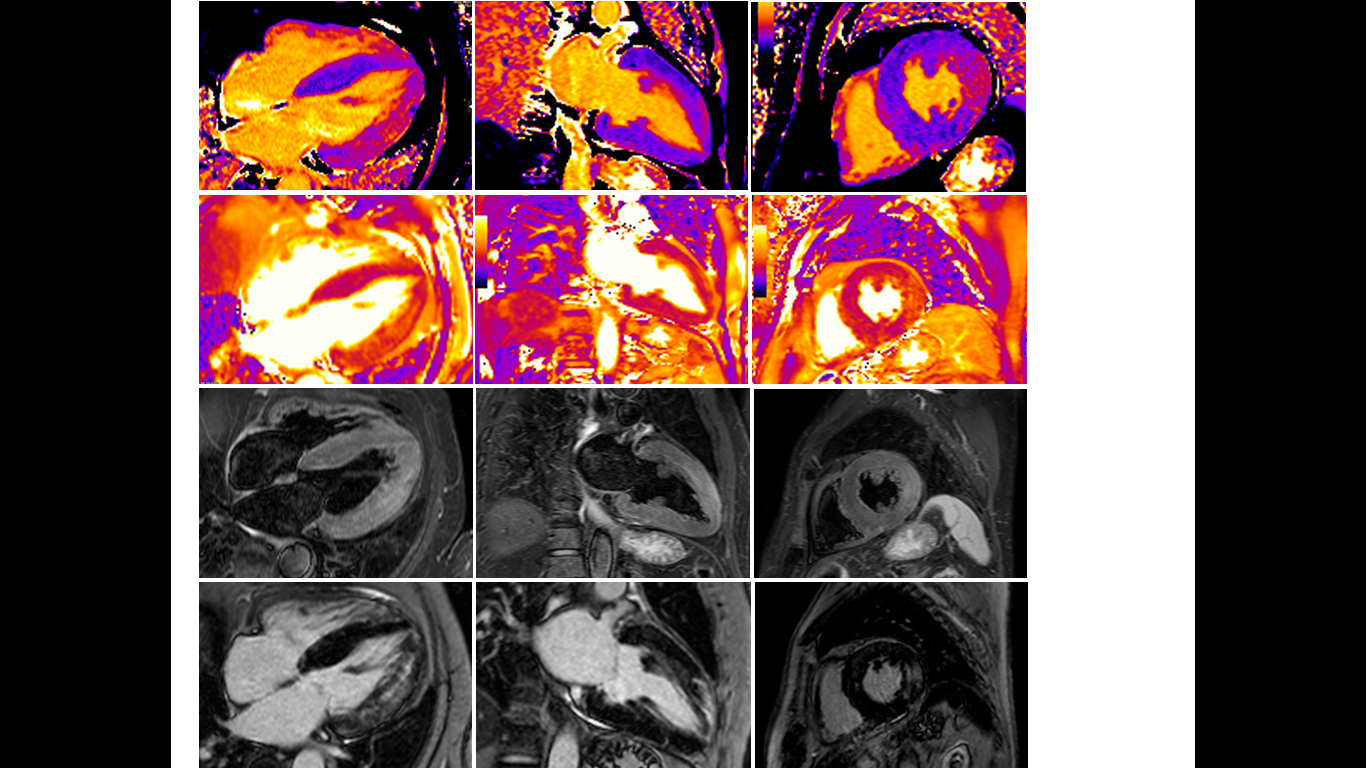


Figure 2: 4-, 2-chambers and mid-short axis view. Panel A: T1 mapping. Panel B: T2-mapping. Panel C: T2-weighted images. Panel D: Motion-corrected segmented inversion-recovery and phase-sensitive-inversion-recovery gradient-echo sequence late-gadolinium enhancement (LGE) imaging. Area of oedema and LGE are highlighted with red arrows.
